# Automatic language analysis identifies and predicts schizophrenia in first-episode of psychosis

**DOI:** 10.1038/s41537-022-00259-3

**Published:** 2022-06-01

**Authors:** Alicia Figueroa-Barra, Daniel Del Aguila, Mauricio Cerda, Pablo A. Gaspar, Lucas D. Terissi, Manuel Durán, Camila Valderrama

**Affiliations:** 1 Department of Psychiatry, Faculty of Medicine, Universidad de Chile, Santiago, Chile; 2 Biomedical Neuroscience Institute, Santiago, Chile; 3 Millennium Nucleus to Improve the Mental Health of Adolescents and Youths (IMHAY), Santiago, Chile; 4 Translational Psychiatry Laboratory Psiquislab, Faculty of Medicine, Universidad de Chile, Santiago, Chile; 5 Artificial Intelligence Development Department, BiosIntelligence, GrupoBios, Santiago, Chile; 6 Integrative Biology Program, Institute of Biomedical Sciences, Faculty of Medicine, Universidad de Chile, Santiago, Chile; 7 Center for Medical Informatics and Telemedicine, Faculty of Medicine, Universidad de Chile, Santiago, Chile; 8 Department of Neuroscience, Faculty of Medicine, Universidad de Chile, Santiago, Chile; 9 Laboratory for System Dynamics & Signal Processing, Universidad Nacional de Rosario and CIFASIS, Santa Fe, Argentina

**Keywords:** Schizophrenia, Biomarkers

## Abstract

Automated language analysis of speech has been shown to distinguish healthy control (HC) vs chronic schizophrenia (SZ) groups, yet the predictive power on first-episode psychosis patients (FEP) and the generalization to non-English speakers remain unclear. We performed a cross-sectional and longitudinal (18 months) automated language analysis in 133 Spanish-speaking subjects from three groups: healthy control or HC (*n* = 49), FEP (*n* = 40), and chronic SZ (*n* = 44). Interviews were manually transcribed, and the analysis included 30 language features (4 verbal fluency; 20 verbal productivity; 6 semantic coherence). Our cross-sectional analysis showed that using the top ten ranked and decorrelated language features, an automated HC vs SZ classification achieved 85.9% accuracy. In our longitudinal analysis, 28 FEP patients were diagnosed with SZ at the end of the study. Here, combining demographics, PANSS, and language information, the prediction accuracy reached 77.5% mainly driven by semantic coherence information. Overall, we showed that language features from Spanish-speaking clinical interviews can distinguish HC vs chronic SZ, and predict SZ diagnosis in FEP patients.

## Introduction

Schizophrenia (SZ) is a severe neurodevelopmental psychotic disorder with a lifetime prevalence of 0.7% that causes emotional, behavioral, sensory, psychomotor, and cognitive alterations with a chronic and deteriorating course^1^. It is common, at least in Chile^2^, to require clinical follow-up and the treating team’s combined effort to confirm or rule out the diagnosis. Moreover, in the case of teenagers, it is a process that spans several months or even a year of transition cycling in and out of mental health services.

Among the research lines, an extensive search of potential biomarkers for improving clinical categorization diagnosis has been performed. In this sense, language biomarkers offer a window to understand the thinking in SZ research^3,4^. In general, individuals with SZ have impaired communicative competencies in fluency, verbal productivity, and speech coherence^5,6^. However, these studies have been performed mainly in English-speaking subjects, and they have used different methodologies to assess language competencies, targeting a wide range of language aspects. In this context, recent authors have begun to explore automated English language assessment in communication tasks, which allows the classifying of healthy controls (HC) vs individuals with SZ^7^. However, the use of such a tool remains in the pilot stage^8,9^. The main reasons provided are the need to better understand language assessment methodologies as well as when and why automated language analysis fails. Therefore, three actions could point towards breaking through the pilot stage of computational tools for schizophrenia language analysis: a better understanding of cross-language variations, dissecting multiple levels of discriminative and predictive language feature capabilities, and focusing on clinically relevant tasks.

Given the reported potential of language biomarkers obtained from clinical interviews of people with SZ and considering our pool of unstructured psychiatric interviews in psychotic subjects, we chose three aspects of language according to this setup to differentiate between HC, first-episode psychosis subjects (FEP), and chronic SZ: fluency, verbal productivity, and coherence.

### Verbal fluency

Verbal fluency (VF) is a complex dimension of communication. Crystal and Davy^10^ point out that FV is synonymous with discursive continuity and includes several elements that are part of this continuous discourse, in particular, pauses and hesitations. Noncommunicative pauses are usually recognized as part of formal thought disorders (FTD) in the mental status examination. Crockford and Lesser^11^ have suggested a relationship between neurocognitive impairment and the appearance of pauses (≥2 s) in aphasia. Interestingly, phonological studies of pauses in English-speaking SZ subjects have shown similar results^12^. Figueroa and Martínez^13^ have also described nonfunctional pauses in Spanish-speaking people with SZ, specifically reporting a longer duration of pauses in FEP subjects. So, the speech of individuals with SZ is interrupted due to frequent and more prolonged pauses with the wrong timing and correlated with negative symptoms^14^. In this context, it is not surprising that automatic pause assessment has also been shown to classify English speakers in HC vs SZ groups, but it is still constrained by the English language^15^. More recently, Stanislawski et al.^16^ studied aberrant pauses in clinical high risk (CHR). Another element of VF is word production and utterances per time as proposed by Clemmer^17^, who studied their patterns in SZ.

### Verbal productivity

Verbal productivity (VP) is the ability to utter a number of words and sentences, such as the number of total words and different words per sentence, average word length, and determiner or pronoun count. In SZ, a low VP, so-called *poverty of speech*, is considered one of the inherent language characteristics in the linguistic profile of SZ patients^18^. In fact, differentiation between HC vs SZ patients^19^ and those affected by antipsychotics^20^ has been demonstrated. On the other hand, some VP measurements such as the number of words and different words, either in interview transcripts of an interview or written narratives^21–24^, differentiate subjects at CHR. Finally, automated VP analysis techniques are also being used as predictors in subjects at CHR showing that pronouns and deictics work as predictive markers of SZ, at least for English speakers^22^, and also to explain cognitive deficit variance^25^.

### Semantic coherence

Semantic coherence (SC) consists of the logical organization of meaning in discourse through interrelated linguistic structures. For example, in interviews with people with SZ schizophrenia, conversation topics can abruptly change. Furthermore, in SZ patients, erroneous and lax use of words or expressions affects concordance, referentiality, and therefore, speech comprehension^21,22,26,27^. Moreover, lax speech requires the listener to make an extra effort to understand what the affected person said. Manual linguistic approaches have been proposed to identify SC, for instance, identifying each sentence’s role in the speech^18,28,29^ and computing indexes such as the Communication Disturbance Index^30^. The pioneering work of Elvevåg et al.^31,32^ proposed automated incoherence measurement. Corcoran et al.^22^ proposed the use of latent semantic analysis (LSA) combined with VP measurements to predict psychosis in CHR populations. Other related work^23^ deals with referential cohesion and its relation to semantic coherence. Since it accounts for the semantic relations that maintain the continuity of discourse, referential coherence is a deeper level of spoken or written semantic coherence, as proposed in systemic functional linguistics^33^.

### Language analysis in non-English-speaking groups

In a multilingual context, there are several studies related to schizophrenia in other languages besides English. In Spanish, our group has reported a longer pause duration in the FEP group^13^ and a positive correlation with negative symptoms^14^, the identification of 24 hierarchical candidate language features to automatize^34^, and the loss of integrity and coherence in FEP and SZ subjects^27^. In Italian, Frau et al.^35^ proposed a semiautomated clustering analysis of speech and its correlation with the speech of SZ patients. The novelty of this work is that it sheds light on the variations of language within schizophrenia groups such as SZ, eventually as a way to measure treatment effectiveness. In Dutch, Wouts et al.^36^ proposed the use of a deep-learning transformer model to capture long-distance language relations. The effectiveness of the method is shown for a 3-class classification problem: control, depressed, and psychotic subjects. In Portuguese, Mota et al.^37^ proposed a computational assessment using graph analysis of syntactic coherence for specific tasks (e.g., memory reports of a dream and negative image) and reported that it provides accurate quantification of speech characteristics and a correlation with clinical symptoms. The work by Mota et al.^38^ is applied to distinguish HC, FEP, and SZ and to do a longitudinal analysis of FEP’s diagnosis.

There are multiple reports of language biomarkers with the clinical potential for analyzing SZ communication skills. However, there are not many studies of SZ onset prediction based on the analysis of other languages besides English speakers. In this study, we propose that language biomarker analysis of VF, VP, and SC can be automatized even in unstructured ecological Spanish-speaking interviews. More specifically, the first goal of this study is to use language to automatically distinguish between healthy controls, first-episode psychosis patients and schizophrenic subjects, and our second goal is to predict which FEP patients convert or do not convert to SZ. In order to achieve these aims, we will evaluate 30 automated linguistic features in a sample of Spanish-speaking HC, FEP, and SZ individuals, and then we will measure their stability, diagnostic, and prognosis capacity in SZ. In addition, we assess the relative contribution of clinical, sociodemographic, and linguistic information for classification purposes.

## Results

**Fig. 1:**
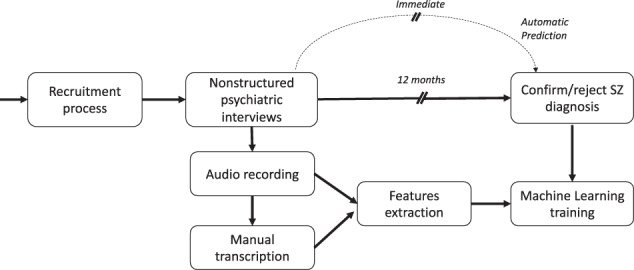
Schema of the data collection process and potential use. Continuous lines indicate information flow and box processes. The dashed line shows a possible benefit.

**Fig. 2:**
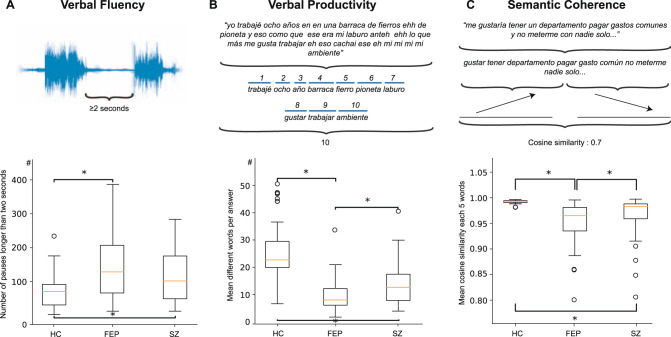
Examples of language features and group comparison. **A** Illustration of pauses longer than two seconds. **B** Example sentence, where stop words are removed and unique words counted. **C** Example measurement of semantic coherence by two five-word-length sentences using cosine similarity. For each feature, the comparison of HC, FEP, and groups is shown. *Statistically significant difference (*P* < 0.001).

One hundred and thirty-three interviews (HC = 49; FEP = 40; and chronic SZ = 44) were recorded and manually transcribed for further automated analysis. The overall data collection process is shown in Fig. 1. HCs were exclusively Spanish-speaking subjects from Chile, without self-reported psychiatric disorders or substance abuse. SZ diagnosis was confirmed by a team of three adult psychiatrists, who used the DSM-IV structured clinical interview^39^, PANSS positive and negative symptom subscales were used for measuring symptom severity of FEP and SZ^40^. FEP was defined as up to two years after presenting their first psychotic episode. At the end of follow-up, 28 FEP subjects confirmed SZ diagnosis (converted to SZ, C-SZ, see Table 2), and 12 transitioned to other nonschizophrenic psychoses (50% transitioned to mood disorders). The full set of 30 language features presented in this study was applied to HC, FEP, and SZ interviews (see Fig. 2 and details in Supplementary Tables S2–S4).

When taking a closer look at the information contributed by each feature, it can be seen that from the 30 evaluated features, 9 clusters of at least two correlated variables (Pearson coefficient) were detected, which provide similar information, as shown in Supplementary Fig. S2. Moreover, sets of correlated variables could be observed; some of them are expected, such as TTR500 grouped with TTR1000 (cluster G in Supplementary Fig. S1) as they represent similar information (type-token ratio) at different text spans. Interestingly, clusters B and C indicate a correlation between word-level features (word length) and sentence features (count of questions–answers). We also looked for associations between language features and symptoms. In the FEP group, two correlations were statistically significant (Pearson, *P* < 0.05): possessive pronouns (*r* = 0.38; *P* = 0.0153), and min cos similarity six levels (*r* = 0.33; *P* = 0.0427). In the SZ group, five measurements were statistically significant: demonstrative and relative pronouns (*r* = −0.49; *P* = 0.007 and *r* = −0.30; *P* = 0.0455, respectively), question–answer pairs per time (*r* = −0.40; *P* = 0.0065), different word per time (*r* = 0.30; *P* = 0.0464), and TTR500 (*r* = 0.32; *P* = 0.0343). Furthermore, in the SZ group pauses were near significant (*r* = −0.29; *P* = 0.0503). Multiple testing Bonferroni correction was applied to above-mentioned correlations (*k* = 30), even though many features are correlated, and only negative PANSS and demonstrative pronoun correlations hold.

### Cross-sectional analysis

**Fig. 3:**
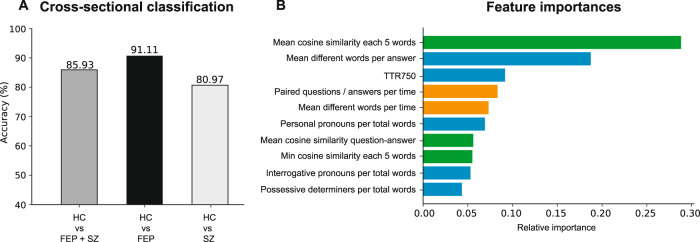
Cross-sectional classification and features. **A** Pairwise Classification accuracy of HC with FEP (black), SZ (light gray), and FEP + SZ (gray). **B** Variable importance list of the HC vs FEP + SZ classification shown in (**A**). Verbal fluency (orange), verbal productivity (blue), and semantic coherence (green) features are listed.

The first goal of this study was to automatically distinguish between subject groups (HC, FEP, SZ) and rank more informative linguistic variables. A variable importance list was compiled using an initial random forest classifier to differentiate between HC, FEP, and SZ subjects, selecting the top 10 most relevant, as shown in Fig. 3B. Using the top ten ranked variables, the accuracies obtained in differentiating between HC and patient groups were 80.97% (HC vs SZ), 85.93% (HC vs FEP + SZ), and 91.11% (HC vs FEP) using a random forest classifier (Fig. 3A).

### Longitudinal analysis

**Fig. 4:**
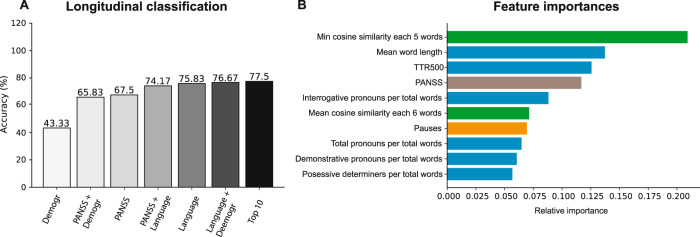
Longitudinal classification and features. **A** Classification accuracy of FEP group into C-SZ and NC-SZ using demographic information (Demogr), PANSS scores, language features, and the top 10 feature selection detailed in (**B**). **B** Variable importance list of the top ten features to classify C-SZ and NC-SZ.

The second goal of this study was to predict which FEP patients convert (C-SZ) or do not convert to SZ (NC-SZ). Our first analysis was similar (correlation analysis is reported in Supplementary Fig. 2) to that of the cross-sectional study: only language variables were used; later, we added clinical (PANSS, duration of disorder) and demographic variables (gender, age, education, first-degree relative with psychotic disorder). Then a new list of top ten features was computed (Fig. 4B). In this ranking, PANSS total score ranked fourth, and all the remaining features were language-related. Compared with the cross-sectional analysis, we observed similar informative features in both scenarios, such as cosine similarity minimum, mean TTR500 and TTR 750, and interrogative/possessive determiners (compare Figs. 3B and 4B).

To evaluate FEP conversion to SZ, we measured accuracy. Using only patient demographic information, results were poor (43.33%), but they improved by using PANSS information (65.83%). PANSS information allowed a 67.5% prediction accuracy. Interestingly, language-only provided 75.83% accuracy. When all information was combined and the top ten features were selected, 77.5% accuracy was achieved to predict if an FEP patient would have a confirmed SZ diagnosis, as shown in Fig. 4A.

**Fig. 5:**
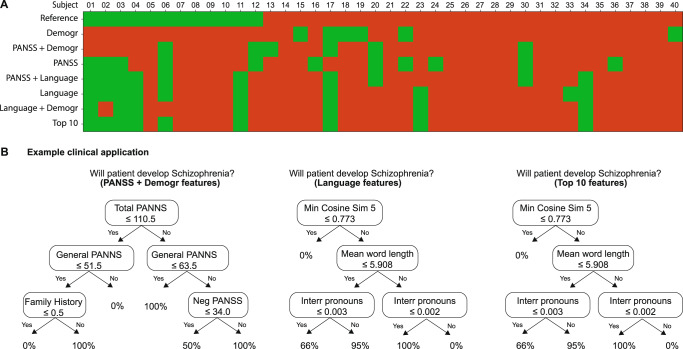
Longitudinal case-by-case analysis and example clinical applications. **A** The first row shows the reference diagnosis for the group of FEP study participants, where NC-SZ is green, and C-SZ is red. Each following row shows classifier performance using a set of features where a classifier match has the same color as the reference diagnosis row. **B** Decision trees illustrate three simplified, automatically generated (three levels) classifiers automatically generated: using clinical features only (PANSS+demographics), language features only, and the top ten selected features in Fig. 4. The top ten features decision tree is identical to language-only features, and only at the fourth level non-language features are used.

A visual report of all FEP 40 patients is shown in Fig. 5A, where the response of all classifiers for the reported feature set selected is displayed. As shown, the demographic information-based classifier overestimated SZ conversion (second row, mainly red). When more language information was included, the classification improved (match of green and red colors with reference). PANNS and language-based classifiers failed to predict six NC-SZ patients’ conversion (subjects 5, 7, 8, 9, 10, 12), which were mainly (5 out 6) affective disorders. In addition, we compared how much each feature category contributes to FEP diagnosis prediction (Supplementary Fig. S3). FEP diagnosis accuracy ranged from 56% (fluency), 64% (verbal productivity), 77% (semantic coherence).

## Discussion

This study expands language biomarkers in SZ and their automated computation, considering non-English speakers and the biomarkers’ overall relation with SZ groups.

### Language markers

In terms of VF, we found that four out of four markers were statistically different between groups (*P* < 0.001). In terms of pauses, it has already been shown that these markers can identify English-speaking HC vs SZ patients^15^, and here we confirmed that the same occurs in Spanish-speaking subjects, even in the case of the FEP group. The total/unique words or total sentences per time also showed differences, which to the best of our knowledge, has not been reported in the literature to date. Moreover, as shown in Supplementary Fig. S1, these features are correlated with productivity markers such as word total mean per answer, giving opportunities for alternative measuring approaches.

Regarding productivity markers, we confirmed that raw volume (total unique words or per answer) or normalized volume (type-token ratio or TTR) could distinguish groups in Spanish, just like in English^20,21^. We also suggest a new productivity marker: mean word length, which can also identify groups. This measurement illustrates the speaker’s greater or lesser linguistic complexity, considering that the frequency of appearance of words in Spanish is concentrated in words composed of one and two syllables (RAE-Corpus CREA) and is calculated by the number of syllables per word.

In the case of syntactic markers, such as the determiners and the pronoun counts, we found that specific pronouns and determiners were different between study groups (see Supplementary Table 3). Previous studies in English^22^ have used syntactic markers such as possessive and interrogative pronouns, reporting a decrease in possessive pronouns in SZ patients. Interestingly, we observed that indefinite pronouns were significantly different (*P* < 0.001), while personal and interrogative pronouns were close to significantly different between groups (*P* < 0.01), as well as indefinite and demonstrative determiners (*P* < 0.01), which may all be related to reduction^41,42^. Referential coherence accounts for the speech functional architecture of speech, and it is known to be altered in individuals with SZ schizophrenia; thus, syntactic markers are a direct and straightforward way to measure this coherence.

Verbal coherence markers has been proposed before in English^22^. We encoded sentences with a different method (word2vec) in our Spanish-speaker database; nonetheless, computing coherence with a span of five or six words can still significantly identify subject groups. We evaluated minimum coherence and mean coherence, and mean values showed more discriminating power, as shown by the *P* value ranking.

Concerning the associations of negative symptoms and language features, in SZ we found a statistically significant VP (TTR500) and VF (question–answer pairs per time, different words per time, and weakly with pauses) as reported by Frau et al.^35^ and Stanivslavsky et al.^16^. Interestingly, in the FEP group, pronouns and semantic coherence (min cos similarity 6 levels) were associated with negative symptoms. Taking into account that PANSS’s negative score was higher in the SZ group (Table 1), we could interpret that previously reported correlations for the poverty of speech and pauses are found with more severe negative symptoms, but lower negative symptom correlations are found only at semantic coherence and specific verbal productivity measurements (possessive pronouns). In the literature, it is reported that semantic alterations are associated with a decrease in the functional connectivity of gamma frequencies, and this alteration is correlated with psychotic symptoms in gchizophrenia^43^. Thus, patterns of semantic alterations and their association with both positive and negative symptoms could shed light on some general mechanisms of functional connectivity alteration.

**Table 1:** Demographics and clinical description of study subjects.

	Healthy control (HC)	First-episode psychosis (FEP)	Chronic schizophrenia (SZ)	*P* value
*n*	49	40	44	—
Gender (% male)	49%	63%	57%	0.43
(% female)	51%	47%	43%	
Age (years)	38.6 ± 15.0	18.1 ± 2.5	35.5 ± 9.3	<0.01
Education (years)	15.2 ± 2.6	10.8 ± 1.7	12.7 ± 2.4	<0.01
Duration of illness: from first episode to interview (years)	—	0.7 ± 0.8	17.5 ± 8.8	<0.01
First-degree relative with psychotic disorder (%yes)	—	50%	52%	0.84
PANSS total score	—	117.7 ± 14.1	145.1 ± 13.1	<0.01
PANSS-positive score	—	26.0 ± 4.9	32.8 ± 4.8	<0.01
PANSS-negative score	—	29.4 ± 5.4	37.9 ± 4.5	<0.01
PANSS general score	—	62.4 ± 7.2	74.4 ± 8.0	<0.01

As shown in Table 1, age among study groups is significantly different, and there are reports of differences between adolescents and older adults (+60) in VF and VP features^44,45^. However, in our study, subjects of age 60 years or older were a very small percentage: 8.1% (4/49) in HC, 0% (0/40) in FEP, and 0% (0/44) in SZ. To further investigate, we compared, in the case of total words per time (VF) and TTR250-500-750-1000 (VP), two linear models with and without the age, and there was no significant difference between models (ANOVA, *P* < 0.05).

### Cross-sectional analysis

Automatic classification of healthy controls vs study participants with schizophrenia shown in this work has up to 80% accuracy using only language-related features, and HC vs FEP has 91.11% accuracy. Thus, we quantitatively demonstrate that distinguishing between HC and SZ is more complex than distinguishing between HC vs FEP, which can be expected since SZ patients are stabilized under regular medication. Literature reports accuracies from 72% (in similar conditions) to 100% for CHR populations^22^. Here, we showed that language analysis has the potential to be used as a psychiatric diagnostic screening tool. In this work, we highlight that many kinds of language biomarkers can solve this problem. Consequently, clinical applications should privilege language independence and ease implementation. In that regard, transcription should be avoided, as language processing is community dependent. For instance, in a Spanish text (from Chilean subjects), we had to create new stop words to perform analyses that are not of everyday use in other Spanish-speaking countries such as Spain or Mexico.

### Longitudinal analysis

To our knowledge, there are no Spanish-speaking studies that predict schizophrenia from the first episode of psychosis. Interestingly, when demographic, PANSS, and language features are combined, higher accuracy is achieved (77%), which may be an indication that these are measuring different aspects of SZ. Furthermore, language biomarkers provide more information than demographic information (75% vs 43% accuracy), and language biomarkers were better than a highly specialized PANSS score (75% vs 67% accuracy). Taking a closer look at the most relevant features to predict SZ onset in FEP subjects (word length, pauses, coherence, pronouns use), according to the ZIPF’s law^46^, in all languages, there is a close relationship between the length of a word and the frequency of occurrence, so longer words are less frequent. According to the RAE (Royal Spanish Academy), in Spanish, there is a high frequency of two-syllable words. We observed a higher occurrence of longer words in participants with SZ, which in general are infrequent words, supporting the findings of several studies^47–49^. On the other hand, the use of short words in interaction with the occurrence of aberrant pauses generates a fragmented speech that is not observed in controls. Likewise, we observed differences in the use of personal and possessive pronouns; it is possible that these findings are clues to referential anomalies in the discourse. We can interpret that TTR, word length, pauses, and determiners are related dysfunctional characteristics of SZ that reduce communication effectiveness, in contrast with HC, and they can contribute to identifying FTD. Overall, our proposed prediction system showed that affective disorders were the most difficult differential diagnosis of SZ, as more prediction errors are accounted for by these subjects. It has been shown that pathologies such as affective disorders show similar formal thought disorders as SZ at an early stage^50^; hence, we can interpret our results as detecting thought disorders that strongly relate to psychosis. Interestingly, our work shows that VF, VP, and SC can predict diagnosis in the case of FEP, as well as a different language aspect such as syntactic coreferences, as proposed by Mota et al.^38^ in a task-specific protocol. A promising perspective is to explore if taken together we can identify more or/and better SZ and other psychosis-related pathologies at the same time.

Neuroimaging biomarkers have also been proposed using structural MRI, EEG, and PET. Kambeitz et al.^51^ performed a meta-analysis evaluating studies that combined neuroimaging techniques and found an overall sensitivity of 80% (CI 77–84%) after evaluating 38 studies. Similarly, Shim et al.^52^ proposed the use of automated EEG analysis to classify between SZ and control subjects, reaching a maximum accuracy of 88.24%. More recently, Zeng et al.^53^ have proposed a deep-learning approach based on MRI, achieving 85% accuracy. However, MRI, PET, and EEG are difficult to apply in clinical settings due to their access, cost, and technical difficulties in low-income countries. In our opinion, language analysis represents an interesting approach that, despite having a lower prediction accuracy, is simpler to apply in medical settings.

We summarize our contributions as (1) a better understanding of cross-language variations. English and Spanish have multiple differences (e.g., longer words are more frequent in Spanish than in English, Zipf law). Thus, it is not evident a priori that the same discriminative or predictive features and methods in English will work in Spanish. One of our results is that most discriminative and predictive language features hold in Spanish for group discrimination, contributing to the understanding of cross-language variations. Furthermore, we can predict diagnosis in FEP, for a small subjects group. (2) Dissecting multiple levels of discriminative and predictive language feature capabilities. To this aim, we compared how much each feature category contributes to the classification of three groups (HC, FEP, SZ) and FEP diagnosis prediction (Supplementary Fig. S3). Interestingly, group classification and FEP diagnosis accuracy are higher for semantic coherence. We argue that more operational tasks such as VF and VP can be impaired differently among subjects. Still, their speech effectiveness is finally affected, and this is more related to semantic coherence. This hypothesis is consistent with our results that rank the semantic coherence dimension as more informative than FV or VP. In this sense, our findings support the proposals of Hinzen and Roselló^41^, who hypothesize that alterations in linguistic cognition may cause alterations in thinking in schizophrenia. An example of these alterations in linguistic cognition is the loss of meta-reflexive abilities derived from higher thought processes, implying a significant impairment of semantic coherence that integrates the selective mechanisms guided by linguistic cognition. (3) Focusing on clinically relevant tasks. Proposed works^24,38^, use psychiatric interviews, where participants are asked to perform a communicative task such as narrating a dream or anecdote. This interviewer-modeled discourse elicitation provides a different communicative framework than the clinical phenomenological interview we used for this study. In the phenomenological interview, discourse elicitation is not determined by a task but follows a natural course of interaction.

### Limitations

This study also has some limitations. First, HCs were exclusively Chilean Spanish speakers, and comorbidities like drug abuse were self-reported. Second, healthy and psychotic recruited subjects had different demographic variables, which could be a potential bias. Third, there was no register of refusals at recruitment. Fourth, the chosen predictive method (random forest) has a relatively simple and broad interpretation. Finally, we used limited samples, which may lead to overfitting, and the longitudinal analysis classes were unbalanced.

## Conclusion

In this work, we determined which information is language-independent and concluded that linguistic phenomena are broadly invariant, with a few exceptions that must be carefully considered, such as syntactic features (determiners, pronouns). In addition, we performed automated language analysis and combined it with clinical information using machine learning techniques; these procedures have achieved classification results comparable to neuroimaging or EEG methodologies, but they have the significant advantage of being easy to apply in a clinical context. To our knowledge, this is the first time that automated language analysis, using unstructured clinical interviews with open-ended questions, has been used in non-English-speaking countries to classify and predict SZ.

## Methods

### Participants

The HC interviews were selected from the ESECH’s study^54^, which consists of the construction of a corpus of more than 300 interviews with neurotypical native speakers of Chilean Spanish. The duration of HC interviews ranged from 32 to 83 min (53.5 ± 10.2 min) with open-ended questions. The data were organized according to the sociodemographic characteristics of the speakers, selecting subjects with ages and education levels similar to those in the chronic SZ group (Table 1). FEP and SZ subjects were recruited from Barros Luco Trudeau Clinical Hospital (CABL). Psychiatric interviews ranged from 5 to 102 min (mean 28.6 ± 16.5 min), depending on the patient’s. All the interviews were conducted with clinically compensated patients. Among the FEP group, three subjects (7.5%), and among the SCZ group, six subjects (13.6%) were hospitalized at the time of the study. Thus, 89.2% (9/84) were receiving outpatient treatment in a mental health service. Substance use was self-reported, and within the FEP group, 20% of subjects (8/40) reported cannabis or alcohol use (3 females, 5 males). In the FEP group, 7.5% of subjects initiated FEP due to substance use (3/40).

Clinical information used for further analysis were age (years), education (years), disease duration (years), and clinical history of psychiatric disorder in first-degree relatives (yes or no) as shown in Tables 1 and 2. Each patient read and signed an informed written consent form, and the protocol was authorized by the “Comité ética científico del Complejo Asistencial Barros Luco” local committee (ID 155). See Supplementary Methods for more information.

**Table 2:** Demographic and clinical description of FEP follow-up groups.

	Converted to SZ (C-SZ)	Not converted to SZ (NC-SZ)	*P* value
*n* total = 40	12	28	—
Gender (%male)	50%	68%	0.29
Age (years)	17.8 ± 2.4	18.2 ± 2.5	0.69
Education (years)	10.7 ± 2.1	10.9 ± 1.6	0.71
Duration of disorder (years)	0.6 ± 0.8	0.7 ± 0.8	0.64
First-degree relative with psychotic disorder (% yes)	58%	46%	0.49
PANSS total score	114.5 ± 15.7	119.1 ± 13.5	0.35
PANSS-positive score	24.1 ± 6.7	26.8 ± 3.7	0.10
PANSS-negative score	27.9 ± 5.7	30.0 ± 5.2	0.27
PANSS general score	62.5 ± 9.5	62.3 ± 6.2	0.93

### Speech processing

The pauses were determined when the temporal separation between two consecutive speech segments was longer than 2 s. Since audio signals had different recording qualities, a noise reduction algorithm was used before pause detection (see details in Supplementary Methods). For text processing, all punctuation marks, phonetic transcription, expression sounds, onomatopoeias, and stop words were eliminated, while words were lemmatized. Stop words were extended with 73 typical Chilean expressions that fit the definition of the stop word (see details in Supplementary Methods).

To improve the performance of classification methods^55,56^, words were codified in high dimension and then into the classifier, consistent with the notion that the meaning of a word depends on the context of neighboring words. To this aim, we used the word2vec algorithm available as an Open Source software package for Python^57^, building a word model specifically for Chilean Spanish (see Supplementary Methods).

### Linguistic features and speech analysis

An individual’s verbal fluency was assessed using the number of pauses longer than two seconds at any time during the interview, as shown in Fig. 2A. As an additional measurement of verbal fluency, we propose the measurement of the number of paired questions–answers divided by the time or duration of the interview, the number of total words, and different words by the hour. Supplementary Table S2 shows the list of verbal fluency features.

Twenty measurements of verbal productivity were analyzed through four approaches: lexical volume (number of total words and different words per answer), type-token ratio (TTR), the average length of words, and count of determiners or pronouns in two variants: total number of words and non-repeated words, both normalized by the number of responses during the interview, and the average per response (see Supplementary Methods).

A total of six semantic measurements were performed. The semantic lexical coherence between sentences (or cosine similarity) was defined from the sum of each of the semantic vectors of the words that compose them between question and answer, and every 5 or 6 words (see Supplementary Methods).

### Statistical methods, variable selection, and classification

The Shapiro–Wilk test was used to check if data were normally distributed. In addition, for each attribute, statistical tests were performed to assess the group’s statistical differences. A Mann–Whitney *U* test was used to compare pairs of groups (HC vs FEP, HC vs SZ, and FEP vs SZ), and a Kruskal–Wallis test was used to compare the three pairs. We used a correlation and random forest analysis for variable ranking^58^ (see details in Supplementary Methods).

## Supplementary information

SUPPLEMENTARY MATERIAL

## Data Availability

The datasets used in this study are not publicly available due to participant privacy and security concerns. Researchers may contact the corresponding author for access.

## Code availability

All the analysis in the work was done in Python, and the code is publicly available at https://github.com/busmangit/nlpezq. The code is organized in Jupyter notebooks and commented on. Please cite this article in case you use the code totally or partially.
