# Supplementary material for: Automatic language analysis identifies and predicts schizophrenia in first-episode of psychosis
Source: Schizophrenia (Heidelb). 2022 Jun 1;8(1):53. doi: 10.1038/s41537-022-00259-3 (PMC9261086; doi:10.1038/s41537-022-00259-3)
Supplement: Supplementary file 1 — SUPPLEMENTARY MATERIAL [file 41537_2022_259_MOESM1_ESM.pdf]

Supplementary Information

Additional methods

First episode and chronic SZ subjects

These interviews belong to the Language, Psychosis and Intersubjectivity project (LEPSI by its initials in Spanish), whose objective is to study linguistic markers for psychosis prediction. LEPSI collects psychiatric interviews of Spanish-speaking Chileans with different psychiatric diagnoses.[1] All patients were stable, receiving oral or depot antipsychotics for at least one month. The patient was invited, prior to their control visit, to participate in the study, which implied only doing an audio recording. It is worth mentioning that the interview was the standard, not any special protocol for the study. We had patients refusing to participate, mainly due to privacy concerns, and in the case of those under 18, the responsible adult authorized the participation.

Audio processing

A preprocessing stage was initially performed to reduce acoustic noise and normalize the signal. In this semiautomatic pre-processing stage, the signal-to-noise ratio was enhanced using spectral subtraction.[2] Later, speech pauses were automatically computed using a voice activity detection algorithm (VAD),[3] which retrieves the segments of speech present in the audio signal.

Text preprocessing

Before the transcripted interviews were analyzed, a preprocessing of the text was done. First, all punctuation marks, phonetic transcription, expression sounds, and onomatopoeias were eliminated, and all text was changed to lower case. These steps were done using regular expression libraries in Python. Then Spanish stop words were obtained from the Natural Language Toolkit (NLTK) package,[4] and extended with Chilean stop words that fit the definition: words that help the reader understand the language but do not alter the meaning of the sentence.[5] Some examples are: “poh /ˈpɔ/,” “weon /we.ˈən/,” “chuta /ˈʧʊ.ta/” (see the complete list in Supplementary Table S1). Lemmatization was done using the pattern package;[6] all the words were lemmatized.

Word embedding

Spanish is distinguished by dialectological marks that generate lexical, intonation, and prosodic differences between Latin America and Spain; therefore, trying to use a standard corpus of text as the raw material for building the word model could lead to biases. To address this issue, we made the word2vec model using HC interviews to train the algorithm directly with Chilean regional Spanish texts. Forty-four additional HC interviews also from Núñez & González[7] were also included only for model training, adding to a total of 93 interviews. Together, the interviews had a volume of about 1.3 million different words (excluding stop words) and 7,072 unique words.

The minimum word count in the corpus for incorporating it into the word2vec model was 3, reducing the risk of adding words with spelling errors into the model. The context window was set to seven, which means word2vec models take seven words before and after the selected word to do word embedding. Each word is represented by a 300 dimension vector, which was reduced from 7,072 to 300 dimensions.

Verbal Productivity

TTR was computed as the number of different words on the total number of words in a written or spoken text of 250, 500, 750, and 1000 total words. The average length of words was computed as the number of characters composing each word in the answers, averaged by the number of words. This analysis was done without lemmatization of the text and excluding stop words. We also calculated the number of pronouns and determiners along with all the answers. Figure 2B illustrates the quantification of the total for different words per answer. Supplementary Table S3 shows the list of verbal productivity features.

Semantic coherence

Cosine similarity is a numerical representation of the coherence between two words or sentences. It is obtained by calculating the cosine of the angle between two vectors. Similar words have vectors that point towards the same direction so that the angle will be close to zero and, thus, the cosine will be near 1 (Figure 2C). Opposite vectors, produced by words or sentences with different meanings or incoherent ones, will have a cosine of -1. Two different approaches were performed to measure the semantic lexical coherence (cosine similarity): coherence between question and answer, and coherence every 5 or 6 words in the answer. Supplementary Table S4 shows the list of semantic coherence features.

Classification

The random forest analysis was performed using the scikit-learn package for Python.[8] The adjusted parameters were 2000 estimators (decision trees) and a maximum depth of 10 for the variable ranking. After ranking the 30 variables and removing correlated ones by keeping the highest ranking within each cluster, the top 10 variables were selected.

To classify into clinical classes (HC, FEP, or SZ) and record for the evolution of the psychosis (Conversion to SZ vs other psychiatric diseases) random forest was also used. The adjusted parameters for random forest were also: 2000 estimators (decision trees) and a maximum depth of 10.

Supplementary Table S1: List of 73 additional Spanish stop words and expressions

"pos", "po", "pués", "pues", "cachai", "cachái", "cachay", "cacháy", "puta", "chuta", "pucha", "oye", "wea", "hueon", "hueón", "weon", "weón", "webon", "webón", "webones", "hueones", "weones", "wevon", "wevón", "wevones", "webona", "weona", "huevón", "huevones", "bakan", "bacan", "bakán", "bacán", "bakanes", "bacanes", "ok", "okey", "okei", "ya","yap", "ser" "e", "ee", "es", "ees", "ems", "em", "eem", "m", "sm", "os", "esm", "eesm", "ms", "as", "ja", "bu", "n", "ay", "us", "uy", "quee", "pa", "uff", "oy", "pum", "ym", "uhum", "usm", "msm", "mjm", "mjum", "aha"

Supplementary Table S2: Verbal fluency features

#	Language feature	HC	FEP	SZ	p-value (3 groups)
01	Pauses per hour	71.11±46.34	137.72±88.73	121.39±79.10	p<0.001
02	Total words per hour	121.24±24.36	67.06±29.02	81.97±28.68	p<0.001
03	Total different words per hour	20.03±2.45	15.16±6.73	18.13±5.28	p<0.001
04	Total question-answer per hour	3.49±1.48	6.05±2.07	5.01±1.97	p<0.001

Supplementary Table S3: Verbal productivity features

#	Language feature	HC	FEP	SZ	p-value (3 groups)
05	Mean total words per answer	25.00±9.96	9.57±5.61	13.71±7.45	p<0.001
06	Mean different words per answer	42.16±22.36	13.44±11.00	20.83±14.73	p<0.001
07	Different words each 250 words (TTR250)	0.48±0.04	0.43±0.04	0.45±0.05	p<0.001
08	Different words each 500 words (TTR500)	0.39±0.02	0.33±0.04	0.36±0.04	p<0.001
09	Different words each 750 words (TTR750)	0.34±0.02	0.29±0.04	0.31±0.04	p<0.001
10	Different words each 1000 words (TTR1000)	0.31±0.02	0.27±0.03	0.29±0.04	p<0.001
11	Mean word length	4.13±0.13	4.02±0.23	4.10±0.20	No
12	Mean word length removing stop words	5.64±0.27	5.25±0.52	5.49±0.42	p<0.001
13	Pronouns per total words	0.24±0.020	0.25±0.03	0.24±0.03	No
14	Personal pronouns per total words	0.11±0.011	0.12±0.03	0.13±0.0232	p<0.01
15	Interrogative pronouns per total words	0.0027±0.0020	0.0058±0.0047	0.0050±0.0043	p<0.01
16	Demonstrative pronouns per total words	0.0150±0.0040	0.0171±0.0063	0.0161±0.0063	No
17	Indefinite pronouns per total words	0.0402±0.0060	0.0382±0.0113	0.0347±0.0061	p<0.001
18	Possessive pronouns per total words	0.0006±0.0005	0.0010±0.0011	0.0009±0.0008	No
19	Relative pronouns per total words	0.0673±0.0171	0.0645±0.0188	0.0620±0.0163	No
20	Determiners per total words	0.0583±0.0070	0.0551±0.0112	0.0551±0.0090	No
21	Interrogative determiners per total words	0.0017±0.0014	0.0026±0.0031	0.0027±0.0028	No
22	Indefinite determiners per total words	0.0360±0.0053	0.0322±0.0098	0.0314±0.0068	p<0.01
23	Possessive determiners per total words	0.0121±0.0038	0.0140±0.0062	0.0140±0.0069	No
24	Demonstrative determiners per total words	0.0086±0.0027	0.0062±0.0039	0.0071±0.0037	p<0.01

Supplementary Table S4: Semantic coherence features

#	Language feature	HC	FEP	SZ	p-value (3 groups)
25	Mean cosine similarity each 5 words	0.99±0.0034	0.95±0.0434	0.9658±0.0398	p<0.001
26	Mean cosine similarity each 6 words	0.99±0.0046	0.95±0.0420	0.9566±0.0591	p<0.001
27	Mean cosine similarity between question-answer	0.79±0.0218	0.77±0.0240	0.7650±0.0275	p<0.001
28	Minimum cosine similarity each 5 words	0.83±0.0807	0.83±0.0580	0.8229±0.0821	No
29	Minimum cosine similarity each 6 words	0.83±0.0751	0.85±0.0573	0.8274±0.0850	No
30	Minimum cosine similarity between question-answer	0.46±0.0878	0.43±0.0763	0.4404±0.0922	No

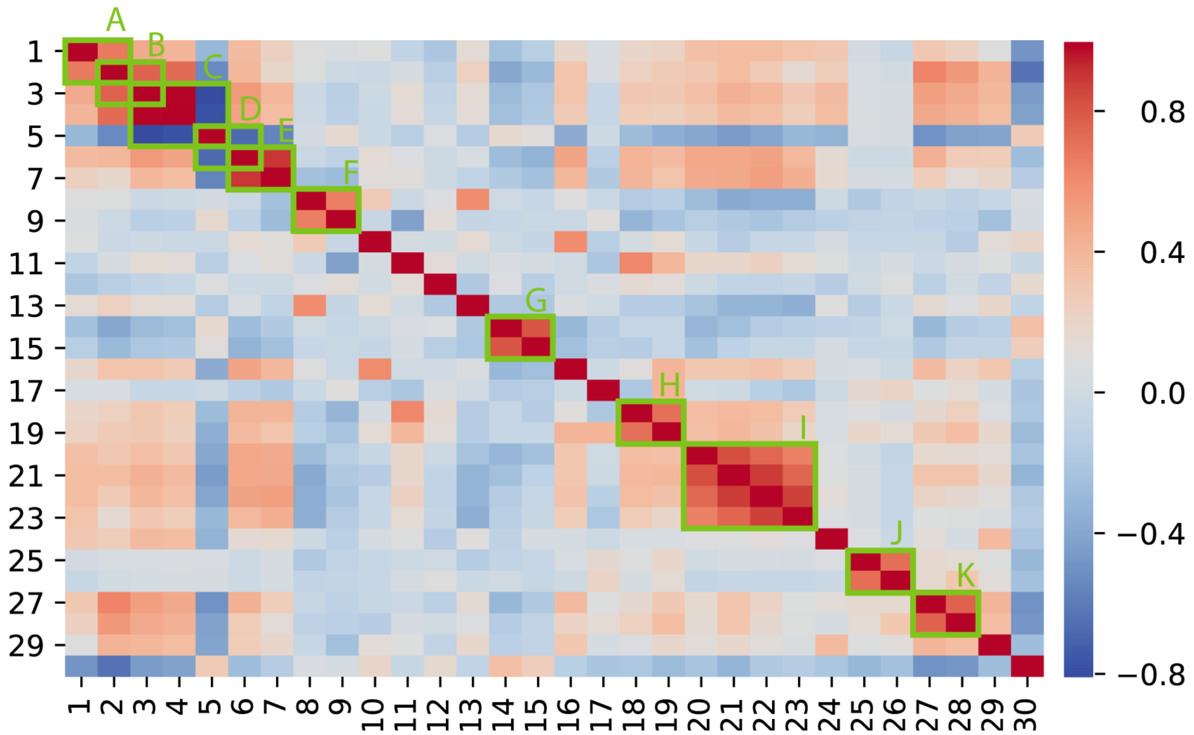

Supplementary Fig. S1. Variable correlation identification for 3 groups. Correlated variables identified by an absolute Pearson's coefficient >= 0.65. Identified clusters are: A Palabras_Diferentes_Por_Tiempo, Palabras_Totales_Por_Tiempo B: Palabras_Totales_Por_Tiempo, Promedio_Palabras_Diferentes_Por_Respuesta C: Promedio_Palabras_Diferentes_Por_Respuesta, Promedio_Palabras_Totales_Por_Respuesta, Pares_Preg-Rpta_Por_Tiempo D: Pares_Preg-Rpta_Por_Tiempo, Largo_Promedio_de_Palabras_Sin_SW E: Largo_Promedio_de_Palabras_Sin_SW, Largo_Promedio_de_Palabras F: Pronombres_Totales_Por_Palabras_Totales, Pronombres_Personales_Por_Palabras_Totales G: Pronombres_Interrogativos_Por_Palabras_Totales, Determinantes_Interrogativos_Por_Palabras_Totales H: Determinantes_Indefinidos_Por_Palabras_Totales, Determinantes_Totales_Por_Palabras_Totales I: TTR250, TTR500, TTR750, TTR1000 J: Minimo_CosSim_5Level, Minimo_CosSim_6Level K: Promedio_CosSim_5Level, Promedio_CosSim_6Level

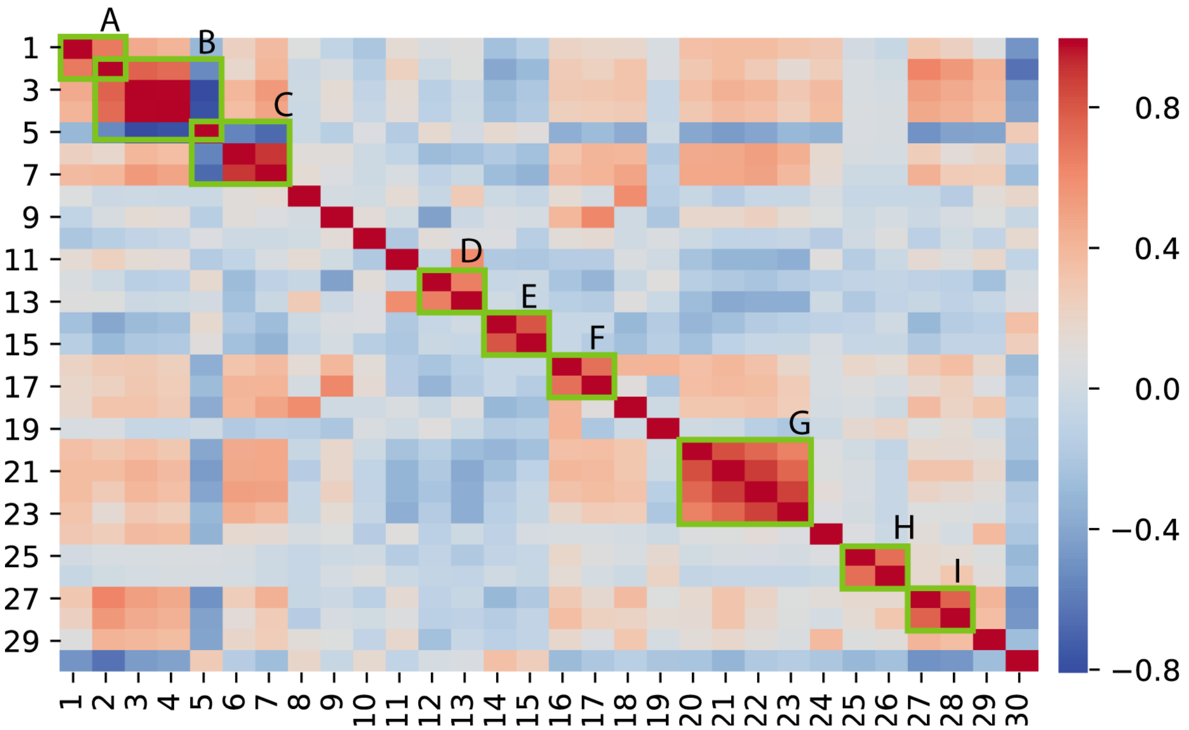

Supplementary Fig. S2. Variable correlation identification for PEE. Correlated variables identified by an absolute Pearson's coefficient >= 0.65. Identified clusters are: A Palabras_Diferentes_Por_Tiempo, Palabras_Totales_Por_Tiempo, B Palabras_Totales_Por_Tiempo, Promedio_Palabras_Diferentes_Por_Respuesta, Promedio_Palabras_Totales_Por_Respuesta, Pares_Preg-Rpta_Por_Tiempo, C Pares_Preg-Rpta_Por_Tiempo, Largo_Promedio_de_Palabras, Largo_Promedio_de_Palabras_Sin_SW D Pronombres_Personales_Por_Palabras_Totales, Pronombres_Totales_Por_Palabras_Totales E Pronombres_Interrogativos_Por_Palabras_Totales, Determinantes_Interrogativos_Por_Palabras_Totales F Determinantes_Totales_Por_Palabras_Totales, Determinantes_Indefinidos_Por_Palabras_Totales, G: TTR250, TTR500, TTR750, TTR1000 H: Minimo_CosSim_5Level, Minimo_CosSim_6Level I: Promedio_CosSim_5Level, Promedio_CosSim_6Level

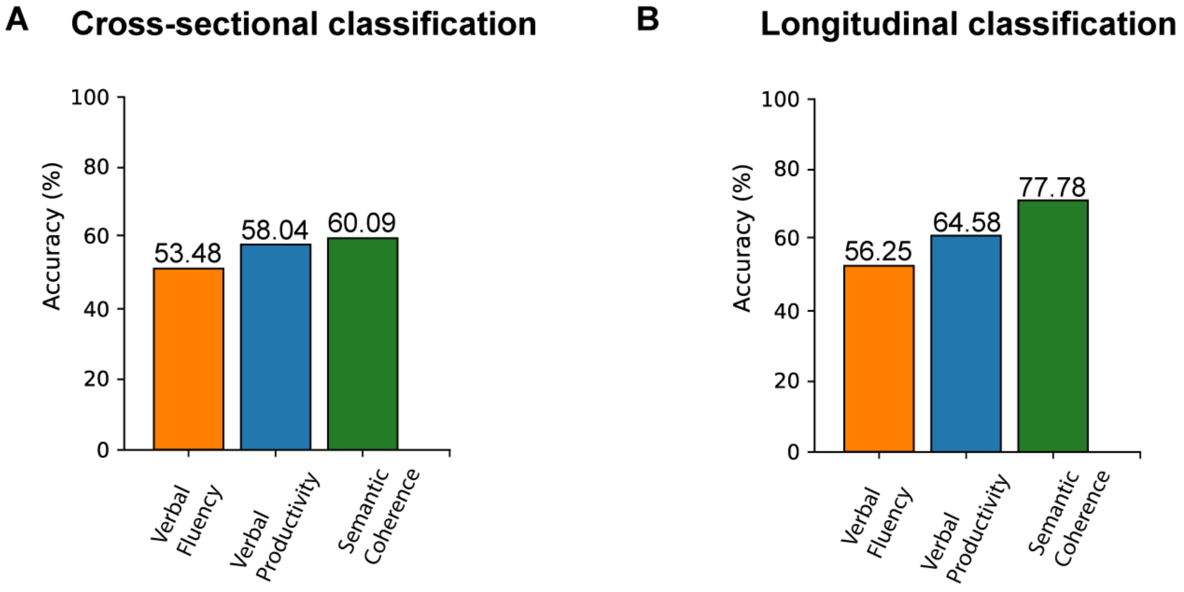

Supplementary Fig. S3. Contribution of language feature categories to classification. A Contribution in the 3-class cross-sectional classification. B. Contribution in the 2-class FEP to SZ conversion classification.

Supplementary References

1. Figueroa, A. Análisis pragmalingüístico de los marcadores de coherencia en el discurso de sujetos con esquizofrenia crónica y de primer episodio. (Universidad de Valladolid, 2015).
2. Boll, S. Suppression of acoustic noise in speech using spectral subtraction. *IEEE Transactions on Acoustics, Speech, and Signal Processing* **27**, 113–120 (1979).
3. Ramírez, J., Segura, J. C., Benítez, C., de la Torre, Á. & Rubio, A. Efficient voice activity detection algorithms using long-term speech information. *Speech Communication* **42**, 271–287 (2004).
4. Loper, E. & Bird, S. NLTK: the Natural Language Toolkit. in *Proceedings of the ACL-02 Workshop on Effective tools and methodologies for teaching natural language processing and computational linguistics* - vol. 1 63–70 (Association for Computational Linguistics, 2002).
5. Hao, L. & Hao, L. Automatic Identification of Stop Words in Chinese Text Classification. in *2008 International Conference on Computer Science and Software Engineering* 718–722 (IEEE, 2008).
6. De Smedt and Walter Daelemans, T. Pattern for Python. *J. Mach. Learn. Res.* **13**, 2063–2067 (2012).
7. Núñez, A. S. M. & González, S. G. Estudio Sociolingüístico del Español de Chile (ESECH): recogida y estratificación del corpus de Santiago. *Boletín de filología* **50**, 221–247 (2015).
8. Pedregosa, F. *et al.* Scikit-learn: Machine Learning in Python. *J. Mach. Learn. Res.* **12**, 2825–2830 (2011).
